# Prevalence of susceptibility patterns of opportunistic bacteria in line with CLSI or EUCAST among *Haemophilus parainfluenzae* isolated from respiratory microbiota

**DOI:** 10.1038/s41598-020-68161-5

**Published:** 2020-07-13

**Authors:** Urszula Kosikowska, Sylwia Andrzejczuk, Ewelina Grywalska, Edyta Chwiejczak, Stanisław Winiarczyk, Dorota Pietras-Ożga, Dagmara Stępień-Pyśniak

**Affiliations:** 1 Department of Pharmaceutical Microbiology, Medical University of Lublin, Chodźki Str. 1, 20-093 Lublin, Poland; 2 Department of Clinical Immunology, Medical University of Lublin, Chodźki Str. 4a, 20-093 Lublin, Poland; 3 Department of Epizootiology and Clinic of Infectious Diseases, University of Life Sciences of Lublin, Głęboka Str. 30, 20-612 Lublin, Poland; 4 Sub-Department of Veterinary Prevention and Avian Diseases, University of Life Sciences of Lublin, Głęboka Str. 30, 20-612 Lublin, Poland

**Keywords:** Infection, Infectious-disease diagnostics

## Abstract

The application of CLSI and EUCAST guidelines led to many discrepancies. Various doubts have already appeared in preliminary stages of microbiological diagnostics of *Haemophilus* spp. A total of 87 *H. parainfluenzae* isolates were obtained from throat or nasopharyngeal swabs from adults 18 to 70 years old, both healthy volunteers and patients with chronic diseases between 2013 to 2015 in eastern Poland. *Haemophilus* spp. were identified by colony morphology, Gram-staining, API NH and MALDI-TOF MS technique. Both susceptibility to various antimicrobials and phenotypes of *Haemophilus* spp. resistance to beta-lactams were determined. Statistically significant association between applied guidelines and drug resistance patterns were observed to as follows: ampicillin, cefuroxime, cefotaxime, amoxicillin-clavulanate, azithromycin, tetracycline and trimethoprim-sulfamethoxazole. Resistance phenotypes according to CLSI vs. EUCAST were as follows: 3.4% vs. 8.0% for BLNAR and 6.9% vs. 19.5% for BLPACR isolates. In conclusion, this is the first study that reports comparative analysis of drug susceptibility interpretation using CLSI and EUCAST of haemophili rods from human respiratory microbiota in Poland. In case of susceptible, increased exposure (formerly intermediate) category of susceptibility within *H. parainfluenzae* isolates we have observed EUCAST as more restrictive than CLSI. Moreover, BLNAI and BLPAI phenotype isolates have been observed, as well as BLPBR using only CLSI or EUCAST guidelines, respectively.

## Introduction

Recently, attention of many researchers is focused on microbes creating human microbiota, their mutual relationships and influence on host’s health^1–3^. Gastrointestinal microbiota is still the most thoroughly examined. Nevertheless, numerous researchers continuously working on other human systems (e.g. respiratory tract), focused on colonizing microorganisms and their potential relevance for host^1,3–5^. Many factors, e.g., way of delivery, having siblings, diet, chronic or recurrent diseases, viral infections or taking various medications, may possibly contribute to the biodiversity, balance, qualitative or quantitative composition of respiratory microbiota^1,3–7^. A frequent and dynamic changes in these microbiota, leading to increased number of infections, including lung and bronchioles diseases in children take abovementioned factors into consideration^5^. Mucous membranes of the upper and lower respiratory tract are recognized as natural environment for numerous commensal microorganisms and pathogens that compose respiratory microbiota such as *Haemophilus* spp., including *Haemophilus influenzae* and *H. parainfluenzae*. The occurrence of such bacteria was found among ten dominant bacterial species in the nasopharynx of young children, where they constituted a specific core of the local microbiota^1^. Haemophili rods may be specific component^1^ or biomarkers of respiratory microbiota^4,6–8^ in people of various age groups and different health states, especially in some chronic diseases. They can play a part in asymptomatic colonization, as well as in infections undergoing in conditions promoting opportunism. Percentage of children colonized by *H. influenzae* can reach up even above 30%, and its excessive colonization of the newborn's respiratory tract may double the risk of pneumonia, bronchitis and otitis media in the first three years of the child's life^9^. Due to the well documented virulence of *H. influenzae* non-typeable (NTHi) strains, presumably other species of *Haemophilus* genus present in oral cavity may also be pathogenic^10^. Growing evidence of infection caused by these opportunistic microorganisms (a series of chronic or recurrent infections), as well as numerous discrepancies during data interpretation using actual recommendations, and growing resistance against commonly used antimicrobials have made this clear.

For that reason, introduction of *H. influenzae* and *H. parainfluenzae* in the scientific elaborations and recommendations published by the Clinical and Laboratory Standards Institute (CLSI)^11^ in 2006 and by the European Committee on Antimicrobial Susceptibility Testing (EUCAST)^12^ over the period 2009–2013, became a huge milestone in this issue. It made possible to interpret obtained results and take correct therapeutic decisions, especially in case of *H. parainfluenzae* for which the choice of antimicrobials and interpretation manner were analogous to *H. influenzae* criteria^11,12^. It is unfortunate that the application of both guidelines led to many discrepancies in classification of microorganisms for particular groups of sensitivity to various antimicrobials, therapeutic effect change or predicted clinical outcome. This is particularly important given the emergence of resistance to beta-lactam antibiotics among *Haemophilus* spp. Especially on the basis of knowledge about *Haemophilus* spp. bacteria resistance phenotypes and differences in resistance genes presence among opportunistic *H. parainfluenzae* isolates from respiratory microbiota^13^.

The novelty and huge value of this work is the comparison of susceptibility results on the basis of CLSI and EUCAST recommendations. Study was conducted on 87 *H. parainfluenzae* isolates being an opportunistic respiratory microbiota compound recovered from healthy volunteers and patients with chronic diseases in eastern Poland over the period 2013–2015, and similar data for haemophili rods has not been found in the literature so far.

## Results

### Susceptibility categories with CLSI and EUCAST

**Figure 1:**
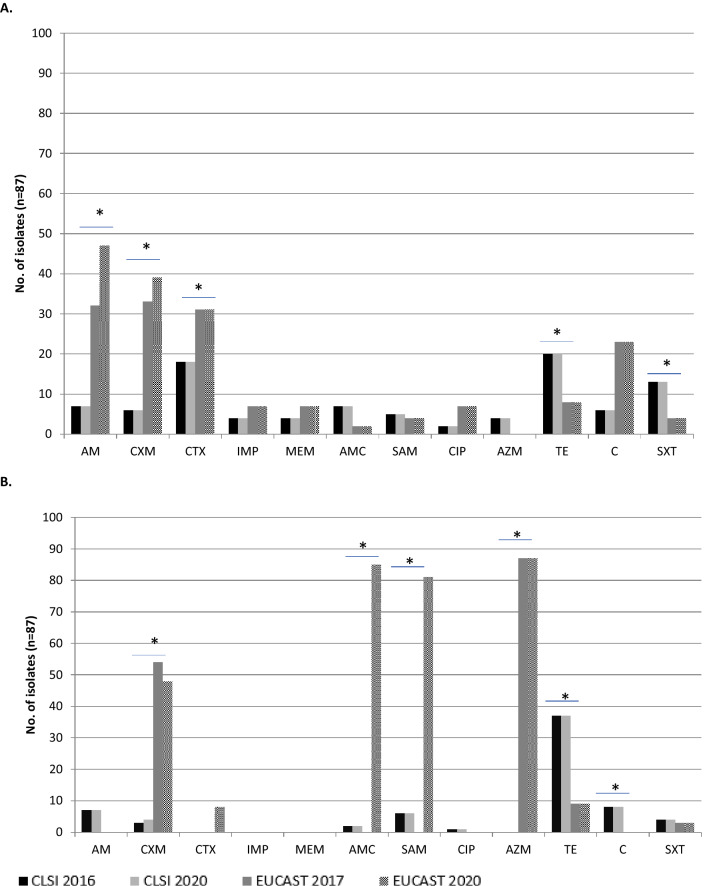
Differences in the number of susceptible, extended exposure (**A**) and resistant (**B**) *Haemophilus parainfluenzae* isolates between CLSI vs. EUCAST recommendations. *p < 0.05, *AMC* amoxicillin-clavulanate, *CTX* cefotaxime, *IPM* imipenem, *MEM* meropenem, *SXT* trimethoprim-sulfamethoxazole, *CXM* cefuroxime, *AM* ampicillin, *TE* tetracycline, *SAM* ampicillin-sulbactam, *AZM* azithromycin, *CIP* ciprofloxacin, *C* chloramphenicol, *CLSI* Clinical and Laboratory Standards Institute, *EUCAST* European Committee on Antimicrobial Susceptibility Testing.

Due to a switching the interpretational criteria from CLSI into EUCAST brought **s**tatistically significant results in categorization of *H. parainfluenzae* isolates tested (Table 1). In the group of sensitive isolates major differences (*p* < 0.0001) have occurred in case of: cefuroxime, amoxicillin-clavulanate, ampicillin-sulbactam and azithromycin, as well as in cefotaxime (*p* = 0.0006) and trimethoprim-sulfamethoxazole (*p* = 0.0216). Isolates categorized as antimicrobials’ resistant differed the most (*p* < 0.0001) in case of as follows: ampicillin, cefuroxime and chloramphenicol, as well as cefotaxime (*p* = 0.0223), tetracycline (*p* = 0.0065) and trimethoprim-sulfamethoxazole (*p* = 0.0144). In Fig. 1 major differences between sensitive, increased exposure and resistant isolates with previous and actual recommendations were presented.

**Table 1:** Differences and statistics between *Haemophilus parainfluenzae* isolates susceptibilities to a various antimicrobials in accordance to CLSI 2016/2020^23,32^ and EUCAST 2017/2020^24,31^ recommendations.

Susceptibility category	Criteria	Antimicrobials No. (%) of isolates (n = 87)											
		AM	CXM	CTX	IMP	MEM	AMC	SAM	CIP	AZM	TE	C	SXT
S	CLSI 2016	73 (83.9)	78 (89.7)	69 (79.3)	83 (95.4)	83 (95.4)	78 (89.7)	76 (87.4)	84 (96.6)	83 (95.4)	30 (34.5)	73 (83.9)	70 (80.5)
	CLSI 2020	73 (83.9)	77 (88.5)	69 (79.3)	83 (95.4)	83 (95.4)	78 (89.7)	76 (87.4)	84 (96.6)	83 (95.4)	30 (34.5)	73 (83.9)	70 (80.5)
	EUCAST 2017	55 (63.2)	0 (0.0)	56 (64.4)	80 (92.0)	80 (92.0)	85 (97.7)	83 (95.4)	80 (92.0)	0 (0.0)	70 (80.5)	64 (73.6)	80 (92.0)
	EUCAST 2020	40 (46.0)	0 (0.0)	48 (55.2)	80 (92.0)	80 (92.0)	0 (0.0)	0 (0.0)	80 (92.0)	0 (0.0)	70 (80.5)	64 (73.6)	80 (92.0)
*p* value		< 0.0001	< 0.0001	0.0006	0.6266	0.6266	< 0.0001	< 0.0001	0.3346	< 0.0001	< 0.0001	0.135	0.0216
I	CLSI 2016	7 (8.0)	3 (3.4)	0 (0.0)	0 (0.0)	0 (0.0)	2 (2.3)	6 (6.9)	1 (1.1)	0 (0.0)	37 (42.5)	8 (9.2)	4 (4.6)
	CLSI 2020	7 (8.0)	4 (4.6)	0 (0.0)	0 (0.0)	0 (0.0)	2 (2.3)	6 (6.9)	1 (1.1)	0 (0.0)	37 (42.5)	8 (9.2)	4 (4.6)
	EUCAST 2017	0 (0.0)	54 (62.1)	0 (0.0)	0 (0.0)	0 (0.0)	0 (0.0)	0 (0.0)	0 (0.0)	87 (100.0)	9 (10.3)	0 (0.0)	3 (3.4)
	EUCAST 2020	0 (0.0)	48 (55.2)	8 (9.2)	0 (0.0)	0 (0.0)	85 (97.7)	81 (93.0)	0 (0.0)	87 (100.0)	9 (10.3)	0 (0.0)	3 (3.4)
*p* value		nd	< 0.0001	nd	nd	nd	< 0.0001	< 0.0001	nd	< 0.0001	< 0.0001	0.0008	0.9605
R	CLSI 2016	7 (8.0)	6 (6.9)	18 (20.7)	4 (4.6)	4 (4.6)	7 (8.0)	5 (5.7)	2 (2.3)	4 (4.6)	20 (23.0)	6 (6.9)	13 (14.9)
	CLSI 2020	7 (8.0)	6 (6.9)	18 (20.7)	4 (4.6)	4 (4.6)	7 (8.0)	5 (5.7)	2 (2.3)	4 (4.6)	20 (23.0)	6 (6.9)	13 (14.9)
	EUCAST 2017	32 (36.8)	33 (37.9)	31 (35.6)	7 (8.0)	7 (8.0)	2 (2.3)	4 (4.6)	7 (8.0)	0 (0.0)	8 (9.2)	23 (26.4)	4 (4.6)
	EUCAST 2020	47 (54.0)	39 (44.8)	31 (35.6)	7 (8.0)	7 (8.0)	2 (2.3)	4 (4.6)	7 (8.0)	0 (0.0)	8 (9.2)	23 (26.4)	4 (4.6)
*p* value		< 0.0001	< 0.0001	0.0223	0.6266	0.6266	0.1187	0.9719	0.1187	nd	0.0065	< 0.0001	0.0144

*AM* ampicillin, *CXM* cefuroxime (oral), *CTX* cefotaxime, *IPM* imipenem, *MEM* meropenem, *AMC* amoxicillin-clavulanate, *SAM* ampicillin-sulbactam, *CIP* ciprofloxacin, *AZM* azithromycin, *TE* tetracycline, *C* chloramphenicol, *SXT* trimethoprim-sulfamethoxazole, *S* susceptible isolates, *I* susceptible, increased exposure (formerly intermediate) isolates, *R* resistant isolates, *CLSI* Clinical and Laboratory Standards Institute; *EUCAST* European Committee on Antimicrobial Susceptibility Testing; *nd* not determined.

### CLSI vs. EUCAST reliance on susceptibility patterns

**Figure 2:**
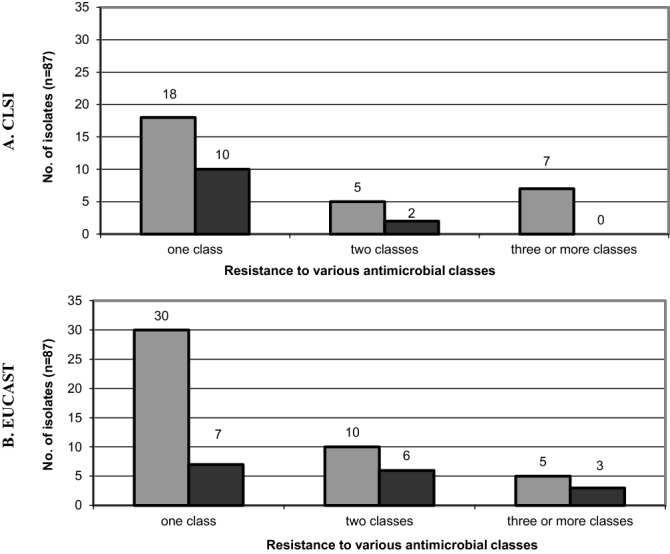
Percentage of *Haemophilus parainfluenzae* resistant to various number of antimicrobials classes**.** *Grey bars* group of isolates selected from patients with chronic diseases, *dark bars* control group of isolates selected from healthy volunteers, *CLSI* Clinical and Laboratory Standards Institute, *EUCAST* European Committee on Antimicrobial Susceptibility Testing.

Table 2 shows susceptibility patterns reliance on applied criteria. We observed that a total of 65.5% (57/87) of *H. parainfluenzae* beta-lactams RPs obtained by both criteria were covered with *p* < 0.001. We have found 48.3% (42/87) vs. 73.6% (64/87) *H. parainfluenzae* isolates resistant to one or more antimicrobials (Table 3) with *p* = 0.0010, respectively. Isolates sensitive to every used antimicrobials at 16.1% (14/87) were not considered in any further analysis. Relevant differences of AST results within two groups of isolates (from control group and chronic disease patients) were revealed (Fig. 2). Among all isolates, 8.0% (7/87) vs. 9.2% (8/87) were MDR strains according to CLSI- vs. EUCAST-derived drug patterns (Fig. 2). With regard to the results among both tested groups of isolates, in control group of healthy volunteers the number of MDR isolates accounted for 8.0% (7/87) vs. 5.7% (5/87), and in a group of chronic diseased patients—0% (0/87) vs. 3.4% (3/87).

**Table 2:** Key practical differences in methodologies between CLSI and EUCAST recommendations for antimicrobial susceptibility testing of *Haemophilus parainfluenzae* isolates by using disk diffusion method.

Recommendation version	CLSI						EUCAST					
	2016			2020			2017			2020		
**Incubation conditions** Temperature Duration Atmosphere	35 ± 2 °C 16–18 h 5% CO_2_						35 ± 1 °C 18 ± 2 h 5% CO_2_					
**Inoculum**	Direct colony suspension, equivalent to a 0.5 McFarland^3^ standard prepared using colonies from an overnight (preferably 20- to 24-h) chocolate agar plate						0.5 McFarland standard					
**Medium**	*Haemophilus* test medium (HTM)						Mueller–Hinton agar + 5% defibrinated horse blood and 20 mg/L β-NAD (MH-F)					
**Interpretive categories and zone diameter breakpoints** (mm)	**Antimicrobial disk contents** (µg; CLSI vs. EUCAST)											
	**S**	**I**	**R**	**S**	**I**	**R**	**S**	**I**	**R**	**S**	**R**	**ATU**
**Azithromycin** (15 µg vs. note)	12	–	–	≥ 12^a^	–	–	note^e^	–	note^e^	note^j^	note^j^	
**Ampicillin** (10 µg vs. 2 µg)	22	19–21	18	≥ 22	19–21	≤ 18	≥ 16	–	< 16	≥ 18^k^	< 18^k^	
**Ampicillin-sulbactam** (10 / 10 µg vs. 10 / 10 µg)	20	–	19	≥ 20^b^	–	≤ 19^b^	note^f, g^	–	note^f, g^	note^k, m^	note^k, m^	
**Amoxicillin-clavulanate** (20–10 µg vs. oral 2–1 µg)	20	–	19	≥ 20^a, b^	–	≤ 19^a, b^	≥ 15^f^	–	< 15^f^	≥ 50^k^	< 15^k^	
**Cefotaxime** (30 µg vs. 5 µg)	26	–	–	≥ 26^c^	–	–	≥ 27	–	< 27	≥ 27^k^	< 27^k^	25–27^l^
**Cefuroxime** (30 µg vs. oral 30 µg)	26	–	–	≥ 20^a, b^	17–19	≤ 16^a, b^	≥ 50	–	< 26	≥ 50	< 27	25–27^l^
**Chloramphenicol** (30 µg vs. 30 µg)	29	26–28	25	≥ 29^c, d^	26–28^p^	≤ 25^c, d^	≥ 28	–	< 28	≥ 28	< 28	
**Ciprofloxacin** (5 µg vs. 5 µg)	21	–	–	≥ 21	–	–	≥ 30	–	< 30	≥ 30^k^	< 30^k^	
**Imipenem** (10 µg vs. 10 µg)	16	–	–	≥ 16	–	–	≥ 20	–	< 20	≥ 20^k, l^	< 20^k, l^	6–19^l^
**Meropenem** (10 µg vs. 10 µg)	20	–	–	≥ 20^c^	–	–	≥ 20	–	< 20	≥ 20^k, n^	< 20^k, n^	
**Tetracycline** (30 µg vs. 30 µg)	29	26–28	25	≥ 29	26–28	≤ 25	≥ 25^h^	–	< 22^h^	≥ 25^h^	< 22^h^	
**Trimethoprim-sulfamethoxazole** (1.25–23.75 µg vs. 1.25–23.75 µg)	16	11–15	10	≥ 16	11–15	≤ 10	≥ 23^i^	–	< 20	≥ 23^i^	< 20^i^	
**Reference strains for quality control**	*Haemophilus influenzae* ATCC 49247 *Haemophilus influenzae* ATCC 49766 *Escherichia coli* ATCC 35218^p^						*Haemophilus influenzae* ATCC 49766 *Staphylococcus aureus* ATCC 29213^r^			*Haemophilus influenzae* ATCC 49247 *Haemophilus influenzae* ATCC 49766 *Escherichia coli* ATCC 35218^p^		

*AM* ampicillin, *CXM* cefuroxime (oral), *CTX* cefotaxime, *IPM* imipenem, *MEM* meropenem, *AMC* amoxicillin-clavulanate, *SAM* ampicillin-sulbactam, *CIP* ciprofloxacin, *AZM* azithromycin, *TE* tetracycline, *C* chloramphenicol, *SXT* trimethoprim-sulfamethoxazole, *S* susceptible isolates, *I* susceptible, increased exposure (formerly intermediate) isolates, *R* resistant isolates,

*CLSI* Clinical and Laboratory Standards Institute; *EUCAST* European Committee on Antimicrobial Susceptibility Testing; *0.5 McFarland standard* bacterial suspension comprising 1–4 × 10^8^ CFU/mL; *NAD* nicotinamide adenine dinucleotide; *ATU* Area of Technical Uncertainty; *ATCC* American Type Culture Collection.

*Notes*^22,31,32^.

^a^Amoxicillin-clavulanate, azithromycin, cefaclor, cefdinir, cefizime, cefprozil, cefuroxime, and clarithromycin used as empiric therapy for *Haemophilus* spp. respiratory tract infections.

^b^Rare BLNAR *H. influenzae* considered as resistant to amoxicillin-clavulanate, ampicillin-sulbactam, cefaclor, cefamandole, cefetamet, cefonicid, cefprozil, cefuroxime, loracarbef, and piperacillin-tazobactam, despite apparent in vitro susceptibility of some BLNAR strains to these agents.

^c^For CSF *H. influenzae*, only results of ampicillin, any of the 3rd-generation cephalosporins, chloramphenicol, and meropenem appropriate to report.

^d^Not routinely reported on urinary tract isolates.

^e^Erythromycin used to determine susceptibility to azithromycin, clarithromycin and roxithromycin.

^f^Benzylpenicillin used to screening, not to distinguish beta-lactamase producing isolates or with PBP mutations.

^g^Susceptibility can be inferred from amoxicillin-clavulanic acid.

^h^Equivalent with susceptibility to doxycycline and minocycline, but some tetracycline resistant may be susceptible to minocycline and/or doxycycline; an MIC should be used if required.

^i^Trimethoprim:sulfamethoxazole in the ratio 1:19; breakpoints expressed as trimethoprim concentration.

^j^Clinical evidence for macrolides efficacy in *H. influenzae* respiratory infections is conflicting due to high spontaneous cure rates; should there be a need to test any macrolide, the epidemiological cut-offs should be used as follows: azithromycin 4 mg/L, clarithromycin 32 mg/L, erythromycin 16 mg/L and telithromycin 8 mg/L.

^k^Benzylpenicillin screen used to exclude beta-lactam resistance mechanisms.

^l^ATU relevant only if benzylpenicillin disk screen is positive.

^m^Susceptibility inferred from amoxicillin-clavulanic acid.

^n^Nalidixic acid test used to screen for fluoroquinolone resistance; nalidixic acid susceptibility can be reported as susceptible to ciprofloxacin, levofloxacin, moxifloxacin and ofloxacin.

^o^Indications other than meningitis.

^p^*Escherichia coli* ATCC 35218—control strain used for testing amoxicillin-clavulanate.

^r^*Staphylococcus aureus* ATCC 29213—control strain for inhibitor component of beta-lactam inhibitor combination disks.

**Table 3:** Comparison of antimicrobial susceptibility patterns of *Haemophilus parainfluenzae* according to CLSI 2016^32^ and EUCAST 2017^31^ recommendations.

Clinical isolate	Susceptibility patterns according to	
	(No. of isolates, n = 87)	
	CLSI 2016	EUCAST 2017
**Group of isolates from healthy volunteers**		
2AU	AmC Ctx Ipm Mem Sxt	Cxm Ctx
2BU	Am Te	Am Cxm Ctx
2CU	Am AmC Sam Ctx	Am Sam Cxm
3CU	Te	–
4AU	Sxt	Ipm
5BU	**Am Te Sxt**	Am Sam Te
6BU	Ctx Te	Cxm Ctx
7AU	Te	Ctx
10AU	–	Ipm
10BU	Am AmC Sam Cxm Ctx Ipm Mem Azm ^11^	Am Sam Cxm Ctx
11AU	AmC Sam Cxm Ctx	Am Cxm
11BU	–	Cxm Ctx Cip
21AU	Mem	–
21BU	–	C
21CU	–	C
22AU	–	Ctx Mem
23BU	–	Am Cxm Ctx C
23CU	Cxm Ctx Ipm	Am Cxm Ctx C
24AU	Sam Ctx Ipm	Am Cxm Ctx Sxt
24BU	**Cxm Ctx C Sxt**	–
24CU	Sxt	–
24GU	–	Am Cxm Ctx C
25CU	**Cxm C Sxt**	Cxm Mem
25BU	Am Ctx	Am Cxm Ctx
26BU	**Cxm Ctx C Sxt**	C
26CU	–	Ctx
27BU	Cxm Ctx	Ctx
27CU	–	Ctx
28BU	–	Ctx
28CU	–	Ipm, C
39CU	–	**Am Cxm Ctx C Sxt**
43AU	**Ctx Azm Te C**	Ctx
46BU	–	–
47BU	–	Ctx
50AU	Sxt	Cxm, C
50CU	–	Am
50DU	Ctx	–
W1HB	Te	Am
W1HC	–	Am
W1HE	–	Am
W2HA	–	Am Cxm Ctx C
W3HA	–	Am Cxm Ctx C
W3HB	Sxt	Am Cxm Ctx
W3HC	–	Sxt
W4HB	AmC Ctx Te	Cxm Ctx Ipm Mem
W4HC	Sxt	**Am AmC Cxm Ctx Ipm Mem Te Cip C**
W5HD	**AmC Azm Te C**	**Am Cxm Te Cip C**
W5HP	Te	**Am AmC Cxm Ctx Te C**
W6HB	–	**Am Cxm Te Cip C**
W7HB	–	C
W7HC	Te	Am Mem
W11HB	**Am AmC Sam Ctx Azm Te C**	–
W12HB	Ctx	Am Cxm Ctx
**Group of isolates from patients with chronic diseases**		
IM 1GB	Te	**Am Cxm Ctx Ipm Mem Te C**
IM 2GB	AmC Ctx	Cxm Cip
IM 4GB	Am	Am
IM 5GB	Mem Te	Cxm C
IM 5GC	–	Am Cxm Ctx C
IM 6GB	Cip	**Cxm Ctx Mem Te Cip C**
IM 6NLB	Sxt	Cxm Ipm Sxt
IM 9GB	Te	Am
IM 9GE	–	Cxm
IM 10GB	–	**Am Cip C**
IM 12NC	–	Cxm
IM 12GB	Te Sxt	Cxm Ctx C
IM 13GB	Te	–
IM 13GC	Te	–
IM 14GC	Cip	Ctx Te
IM 16GB	Sxt	–
IM 18GA	–	Am
IM 18GB	–	Am
IM 20GB	–	Am

*AmC* amoxicillin-clavulanate, *Ctx* cefotaxime, *Ipm* imipenem, *Mem* meropenem, *Sxt* trimethoprim-sulfamethoxazole, *Cxm* cefuroxime, *Am* ampicillin, *Te* tetracycline, *Sam* ampicillin-sulbactam, *bolded patterns* multi-drug resistant (MDR) strains, *Azm* azithromycin, *Cip* ciprofloxacin, *C* chloramphenicol, *CLSI* Clinical and Laboratory Standards Institute, *EUCAST* European Committee on Antimicrobial Susceptibility Testing.

### CLSI vs. EUCAST resistance phenotypes

**Figure 3:**
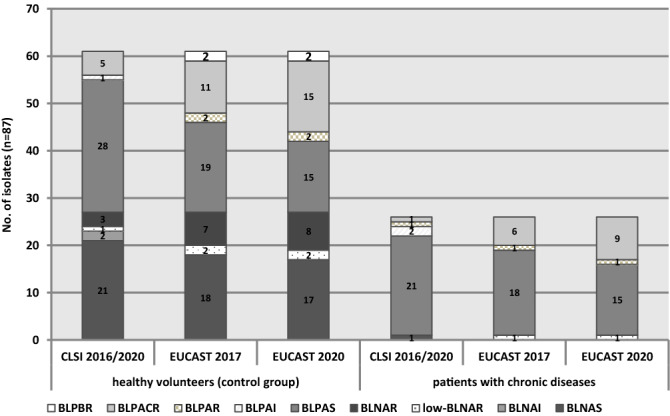
Distribution of resistance phenotypes among *Haemophilus parainfluenzae* isolates. *CLSI* Clinical and Laboratory Standards Institute, *EUCAST* European Committee on Antimicrobial Susceptibility Testing, *BLNAS* beta-lactamase-negative, cefinase-negative susceptible to ampicillin, *BLNAI* beta-lactamase-negative, cefinase-negative, ampicillin intermediate, *low-BLNAR* beta-lactamase-negative, cefinase-negative, ampicillin resistant (MIC_Am_ = 0.5–2.0 mg/L), *BLNAR* beta-lactamase-negative, cefinase-negative, ampicillin resistant (MIC_Am_ ≥ 2.0 mg/L), *BLPAS* beta-lactamase-positive, cefinase-negative, ampicillin and/or amoxicillin susceptible, *BLPAI* beta-lactamase-positive, cefinase-negative, ampicillin intermediate, *BLPAR* beta-lactamase-positive, cefinase-negative, ampicillin and bezylpenicillin resistant, amoxicillin-clavulanate susceptible, *BLPACR* beta-lactamase-positive, cefinase-negative, ampicillin, amoxicillin-clavulanate or benzylpenicillin resistant, *BLPBR* beta-lactamase-positive, cefinase-positive, resistant to one or more beta-lactams (benzylpenicillin, ampicillin, cephalosporins or carbapenems).

According to CLSI 2016, majority of 56.3% (49/87) and 25.3% (22/87) *H. parainfluenzae* isolates were of BLPAS and BLNAS phenotype, respectively (Fig. 3). EUCAST 2017 criteria resulted in majority of BLPAS isolates 42.5% (37/87), followed by BLNAS and BLPACR phenotypes found in 20.7% (18/87) and 19.5% (17/87) of isolates, respectively. Prevalence of RPs among *H. parainfluenzae* isolates occurred CLSI-derived BLNAI and BLPAI phenotype occurred in 2.3% (2/87) and 3.4% (3/87) of isolates, respectively. Phenotype called BLPBR defined as beta-lactamase-positive, cefinase-positive, resistant to one or more beta-lactams (benzylpenicillin, ampicillin, cephalosporins or carbapenems) was observed in 2.3% (2/87) of *H. parainfluenzae* isolates using exclusively EUCAST criteria (Fig. 3). CLSI 2020 criteria did not change anything in our results, whereas EUCAST 2020 increased the number of BLPACR isolates to 27.6% (24/87) and decreased of BLPAS isolates to 34.5% (30/87). Prevalence of RPs among isolates selected from healthy volunteers and patients with chronic disease was performed (Fig. 3). We have revealed a seven various patterns in control group of isolates. Among isolates of the second group, five vs. four RPs of CLSI vs. EUCAST have been found, respectively. In CLSI-derived results among patients with chronic diseases a strong dominance of beta-lactamase-positive phenotypes was assessed.

## Discussion

Reliable interpretation of microorganisms’ sensitivity data to various antimicrobial drugs may create some difficulties and requires expertise. During our studies, association between applied interpretation criteria and susceptibility patterns, as well as different phenotypes of resistance to selected antimicrobials among opportunistic haemophili rods were detected. Many authors^14–20^ point to the differences occurring at the following fields: categorization of drug susceptibility, assignment to individual beta-lactam phenotypes of resistance in *Haemophilus* spp. bacteria, as well as CLSI^21^ and EUCAST^22^ cut-off values. The CLSI 2020 criteria has not changed since 2016, except cefuroxime sensitivity category ranges for this type of bacteria^23^ On the contrary, EUCAST 2020 update brought significant changes in cut-off values, especially for ampicillin and amoxicillin with clavulanic acid for haemophili rods^24^. This resulted in an increase of the number of e.g.: isolates resistant to these antibiotics, and BLPACR phenotype isolation, as well as a decrease in the number of BLPAS phenotype and MDR isolates selection consequently. Among isolates selected from healthy volunteers the number of MDR isolates have been higher regardless of criteria used in comparison with diseased patients group in which, by contast, EUCAST-derived dominance of MDR isolates has been observed.

In this study, we observed both criteria—CLSI and EUCAST coincide with obrained RPs amongst *H. parainfluenzae* (*p* < 0.001). Either CLSI-derived BLNAI and BLPAI or exclusively EUCAST-derived BLPBR phenotype defined as beta-lactamase-positive, cefinase-positive, resistant to one or more beta-lactams (benzylpenicillin, ampicillin, cephalosporins or carbapenems) found in 2.3% of *H. parainfluenzae* isolates, isolates revealed practical disparity. Prevalence of RPs among isolates selected from healthy volunteers revealed greater diversity regardless of guidelines used, whereas among isolates selected from patients with chronic diseases a strong dominance of beta-lactamase-positive phenotypes in CLSI-derived results was assessed.

Key practical differences between each guidelines for disk diffusion method in comparison with the latest ones (EUCAST 2020, CLSI 2020) were performed (Table 2), including both methodologies (e.g., bacteriological media, incubation conditions, antibiotic disc contents, zone diameter breakpoints), as well as reference strains for routine quality control. First doubts have already appeared in the preliminary, microorganisms identification stage. Two types of solid media are in use (HTM, MH-F) for AST. Both contain acidic casein, starch, agar, as well as beef and yeast (HTM) or meat extracts (MH-F), and specific growth factors for *Haemophilus* spp. bacteria—NAD-phosphate (V) and/or hem/haemin/hematin (X)^25^, in the form of HTMS supplement^26^ or 5% mechanically de-fibered horse blood. Many microbiologists preferred to use other media such as MHF or chocolate agar which may support haemophili rods in a better way due to improved nutritive quality, resulting in increased MIC values and decreased growth inhibitory zones^14^. The adoption of richer medium such as chocolate agar or agar enriched with blood may also result in a sudden unwarranted increase of BLNAR strains (from 3.5% on HTM agar, through 14.7% on MHF, to 15.4% on chocolate agar) during agar dilution methods^14,18^. Currently the use of HTM agar as a reference medium in CLSI recommendations is more often questioned^14^.

Introduction of EUCAST can result in significant differences compared to CLSI in classification of microorganisms for particular groups of sensitivity to antimicrobials, being considered as more stringent. Classification of medium-sensitive—intermediate strains became great variability, because EUCAST discarded such category and changed the definition into ‘susceptible, increased exposure’ in 2019. A microorganism is categorized as above when a high likelihood of therapeutic success is observed because exposure to the agent is increased by adjusting the dosing regimen or by its concentration at the site of infection^22^. In practice, CLSI strains in question are usually assigned on the basis of EUCAST to resistant ones. From a clinical point of view, it deprived therapists the possibility of using a damp antimicrobial drug in therapy. Application of stricter criteria and breakpoints in accordance with EUCAST was aimed at reducing the misuse of antibiotics and controlling increasing level of resistance to them^27^. This is an important issue, because different criteria for interpreting the results of antibiograms could have influenced phenotypic determination of resistance among others to beta-lactam antibiotics. It also could be a critical spectra in context of decisions regarding treatment of chronic diseases, in which formation could have been involved e.g. opportunistic microbes resistant to this group of drugs. In addition to therapeutic aspects, another consequence arising from interpretation of received data were either potential epidemiological consequences or the spread of resistance among *Haemophilus* spp. being a respiratory tract microbiota component and other bacteria e.g. to beta-lactams. In our study, among *H. parainfluenzae* isolates switching the criteria from CLSI 2016 into EUCAST 2017 resulted in statistically significant (*p* < 0.05) modifications in (1) sensitivity mostly to beta-lactams with or without inhibitors (ampicillin, cefuroxime, cefotaxime, amoxicillin-clavulanate, ampicillin-sulbactam), as well as macrolides (azithromycin), tetracyclines (tetracycline) and trimethoprim-sulfomethoxazole; (2) susceptibility, increased exposure (formerly intermediate) mainly to cefuroxime (3.4% vs. 62.1%), azithromycin (0% vs. 100%) and tetracycline (42.5% vs. 10.3%); (3) resistance to ampicillin (8.0% vs. 36.8%), cefuroxime (6.9% vs. 37.9%), tetracycline (23.0% vs. 9.2%) and chloramphenicol (6.9% vs. 26.4%). The comparison of our results with the data obtained during the interpretation with the most current recommendations from 2020 revealed several more important problems. Both CLSI reccomendations were consistent with overall isolates categorization. The update caused only slight changes in the number of sensitive and susceptible, increased exposure of isolates to cefuroxime. In turn, the introduction of actual EUCAST 2020 recommendations caused major statistically significant changes, mainly in relation to beta-lactams with or without beta-lactamase inhibitors, as well as microlides and chloramphenicol or trimethoprim-sulfamethoxazole. This, considering all, may suggest significant discrepancies related to a definition of ‘intermediate’ category of isolates which may result in confusion and inaccuracy when prescribing treatment for example to urinary tract infections^20^. Generally, older versions of recommendations may have resulted in a lack of clinical response, while newer versions may reduce or even avoid therapeutic errors or failures.

The most frequently used antibacterial drugs in diseases caused by *H. parainfluenzae*, are beta-lactams used in mono- or in a combination therapy with antimicrobials from other therapeutic classes^12,26^. Analysis of our AST results within two groups of *Haemophilus* spp. isolates either from control group or chronic disease patients revealed relevant differences, especially significant reliance of applied guidelines on drug resistance patterns to e.g.: ampicillin, cefuroxime and cefotaxime. Moreover, susceptibility to amoxicillin-clavulanate of *H. parainfluenzae* depended on applied guideline, in a clavulanic acid dose-unrelated manner. On the contrary, high compatibility for amoxicillin-clavulanate between CLSI and EUCAST can be observed^20^. On the other hand, if EUCAST was used, a higher percentage of resistant *E. coli* and *Klebsiella* spp. isolates as well as lower one of *Proteus* spp. can be obtained^15^. Further, many authors noted significant differences between amoxicillin-clavulanate MIC determination methods. Researchers have come to the final conclusion that EUCAST-derived amoxicillin-clavulanate MIC values were more predictive of therapeutic failure than CLSI ones^15^. Demonstrated differences in the assessment of drug resistance of haemophili rods, both isolated as etiological factors of acute infections^28^, as well as examples of microbiota components shown in our work, may indicate a great need to further research and verification of criteria for AST. It should be taken into account that either the incorrect determination of drug resistance of pathogens or the presence of antimicrobial resistance genes among bacteria derived from microbiota, can play a significant role in the therapy development and therapeutic failure.

In our study, according to CLSI 2016 vs. EUCAST 2017, 48.3% vs. 73.6% of *H. parainfluenzae* isolates were resistant to one or more antimicrobials (*p* = 0.0010). Relevant discrepancies occurred during analysis of both *H. parainfluenzae* isolates groups as follows: distinctly more isolates resistant to one or more antimicrobials classes regardless of considered criteria; in the chronic disease group—nearly two times more isolates resistant to one class and two classes of antimicrobials according to EUCAST; in the control group—no isolates resistant to three or more antimicrobials classes observed using CLSI criteria; and three-folded higher number of such isolates when EUCAST applied. EUCAST beta-lactam interpretative criteria of *Haemophilus* spp. can be considered as close to median MICs for susceptible population, with subtle changes in MICs which slightly influenced on the proportion of strains categorized as resistant at the same time^16,17^. This may also contribute the application of appropriate treatment in patients with infections caused by a microbes producing specific beta-lactamases, either ESBL or AmpC^13,17^.

To conclude, this is the first study that reports comparative analysis of drug susceptibility interpretation using CLSI and EUCAST recommendations of haemophili rods isolated from human respiratory microbiota in Poland. In our study, it was observed EUCAST criteria as more restrictive in case of susceptible, increased exposure category within tested isolates. We have found 65.5% statistically significant covering of resistance phenotypes obtained by both CLSI and EUCAST criteria. Either CLSI-derived BLNAI and BLPAI or EUCAST-derived BLPBR phenotypes were determined. We have also found the EUCAST-derived dominance of MDR isolates amongst isolates from chronic diseased patients.

Prevalence of RPs among isolates selected from healthy volunteers revealed a strong dominance of beta-lactamase-positive phenotypes in CLSI-derived results in *Haemophilus* spp. bacteria. In this study, we observed EUCAST recommendations more restrictive in case of susceptible, increased exposure (formerly intermediate) category within tested haemophilic rods isolates. We agree with many other authors who agreeably suggested a still need of EUCAST breakpoints major revision, as well as a careful analyses and changes in EUCAST criteria, comprising all specialists involved in the therapeutic process of infectious diseases. Both incorrect determination of drug resistance of pathogens and the presence of antimicrobial resistance genes among bacteria derived from human microbiota, can play much more important role in the therapy development and therapeutic failure.

## Methods

### *Haemophilus* spp. isolates

A total of 87 *H. parainfluenzae* isolates were obtained from throat and nasopharyngeal swabs from adults (18–70 years of age) in eastern Poland between 2013 and 2015. Experimental protocol was approved by the ethical standards of the Medical University of Lublin Bioethical Commission No. KE-0254/75/2011 28 April 2011 and prolonged as No. KE-0254/59/2016 25 February 2016. Informed consent was obtained from all individual participants included in the study.

Isolates were subdivided into two separate groups: (1) 61 isolates selected from healthy volunteers as the control group and (2) 26 isolates selected from patients with chronic diseases (either lung cancer or chronic lymphocytic leukaemia). Specimen was collected from cancerous patients before, during and after given chemotherapy. The following reference from the American type Culture Collection (ATCC) were used: *H. parainfluenzae* ATCC 33392, *H. parainfluenzae* ATCC 51505, *H. influenzae* ATCC 10211, *H. influenzae* ATCC 49766, *H. influenzae* ATCC 49247 and *Escherichia coli* ATCC 35218.

### Culture and identification

Isolates were stored as a frozen stock in trypticasein soy broth (TSB, Biocorp, Poland) with *Haemophilus* Test Medium Supplement (HTMS, Oxoid, Hampshire, United Kingdom), in the presence of 30% (v/v) glycerol at − 70 ± 2 °C until its use. Bacteria were then re-cultured by applying a frozen bacterial suspension on chocolate agar (BioMerieux, Craponne, France), incubated for 24 h in microaerophilic (5–10% CO_2_, 80–90% N_2_, 5–10% O_2_, Generbag microaer, BioMerieux, Craponne, France) conditions at 35 °C. *Haemophilus* spp. isolates were identified as previously shown^13^ by colony morphology, Gram-staining and API NH microtests (BioMerieux, Craponne, France), as well as by using the Ultraflextreme Matrix Assisted Laser Desorption Ionization Time of Flight Mass Spectrometry (Bruker Daltonics, Bremen, Germany) (MALDI-TOF MS) with MALDI-Biotyper 3.0 software (Bruker Daltonics, Bremen, Germany) according to the procedure described earlier^29^.

### Antimicrobial susceptibility testing (AST)

The susceptibility to the following antimicrobials: ampicillin (Am), amoxicillin-clavulanate (AmC), ampicillin-sulbactam (Sam), cefuroxime (Cxm), cefotaxime (Ctx), imipenem (Ipm), meropenem (Mem), azithromycin (Azm), tetracycline (Te), chloramphenicol (C) and thrimetoprim-sulfametoxazole (Sxt), was determined by the Kirby-Bauer disk-diffusion method^30^ under the current recommendations (Table 2). Diameters of inhibition of bacterial growth zones around drug discs were measured by using the Interscience Scan^®^ 1200 version 8.0.3.0 (Interscience, Saint-Nom-la-Bretèche, France). All AST experiments and evaluation criteria were performed in accordance with EUCAST 2017 vs. 7.0^31^ and CLSI 2016 26th ed^32^ guidelines and regulations. The most actual EUCAST 2020 vs. 10.0^24^ and CLSI 2020 30th ed^23^ recommendations were also used for comparative purposes.

In a subsequent step, the minimal inhibitory concentrations (MICs) values for ampicillin (MIC_Am_) were determined by the E-test method using the E-test strips (BioMerieux, Craponne, France) with antibiotic at a concentration gradient of 0.016–256 mg/L. Strips were placed on Mueller–Hinton agar with 5% defibrinated horse blood and 20 mg/L β-NAD (MH-F; BioMerieux, Craponne, France) after medium inoculation, incubation was performed in abovementioned microaerophilic conditions for 24 h at 35 °C. MIC_Am_ breakpoints were interpreted according to EUCAST 2017 (susceptible MIC ≤ 1.0 mg/L, resistant MIC > 1.0 mg/L) and CLSI 2016 (susceptible MIC ≤ 1.0 mg/L, intermediate MIC = 2.0 mg/L, resistant MIC ≥ 4.0 mg/L) criteria. According to EUCAST^24^ and CLSI^23^ 2020 guidelines, MIC_Am_ cut-off values have not changed. To determine the beta-lactamase production ability, either benzylpenicillin (1 U, Becton Dickinson, Franklin Lakes, USA) or cefinase test (Becton Dickinson, Franklin Lakes, USA) were used.

### Resistance phenotypes (RPs)

The multidrug-resistance (MDR) was defined as resistance to one or more antimicrobials from three or more therapeutic classes^22^. Phenotypes of resistance to beta-lactam antibiotics for *Haemophilus* spp. were identified on the basis of as follows: cefinase test, ampicillin MIC values, susceptibility to beta-lactams, benzylpenicillin 1U disk test, as well as determination of the presence of selected beta-lactamase *bla* genes described previously^13^. Accordingly, RPs were than classified into nine various categories: beta-lactamase-negative ampicillin sensitive BLNAS, ampicillin intermediate BLNAI, ampicillin resistant low-BLNAR and BLNAR, as well as beta-lactamase-positive ampicillin sensitive BLPAS, ampicillin intermediate BLPAI, ampicillin resistant BLPAR, amoxicillin-clavulanate resistant BLPACR and beta-lactams resistant BLPBR, as described earlier^13^ and below the Fig. 3 in this paper.

### Statistical analysis

Data were summarized and analysed by using GraphPad InStat 3.00 (GraphPad Software, USA). The *p* value (*p* < 0.05 considered as significant), the 95% confidence interval ranges (95% CI) and the relative risk (RR), calculated by the approximation of Katz, Pearson’s Chi-square test or the Fisher’s exact test were used for analysis of the extent of agreement between CLSI and EUCAST.

### Ethical approval

All procedures performed in study involving human participants were in accordance with the ethical standards of the Medical University of Lublin Bioethical Commission No. KE-0254/75/2011 28 April 2011 and prolonged as No. KE-0254/59/2016 25 February 2016. Informed consent was obtained from all individual participants included in the study. This article does not contain any studies with animals performed by any of the authors.

## References

[1] Biesbroek G (2014). Early respiratory microbiota composition determines bacterial succession patterns and respiratory health in children. Am. J. Respir. Crit. Care Med..

[2] Lederberg J (2000). Infectious history. Science.

[3] Quigley EMM (2017). Basic definitions and concepts: Organization of the gut microbiome. Gastroenterol. Clin. N. Am..

[4] Kosikowska U, Biernasiuk A, Rybojad P, Łoś R, Malm A (2016). Haemophilus parainfluenzae as a marker of the upper respiratory tract microbiota changes under the influence of preoperative prophylaxis with or without postoperative treatment in patients with lung cancer. BMC Microbiol..

[5] Koppen IJN, Bosch AATM, Sanders EAM, van Houten MA, Bogaert D (2015). The respiratory microbiota during health and disease: A paediatric perspective. Pneumonia.

[6] Kosikowska U, Rybojad P, Stepien-Pysniak D, Zbikowska A, Malm A (2016). Changes in the prevalence and biofilm formation of *Haemophilus influenzae* and *Haemophilus parainfluenzae* from the respiratory microbiota of patients with sarcoidosis. BMC Infect. Dis..

[7] Kosikowska U (2016). The association of chronic Hepatitis C with respiratory microbiota disturbance on the basis of decreased Haemophilus spp. Colonization. Med. Sci. Monit..

[8] Kosikowska U, Juda M, Malm A, Jozwiakowska M, Tuszkiewicz-Misztal E (2007). Drug susceptibility of Haemophilus sp. and *Staphylococcus aureus* strains colonizing respiratory tract in children with mucoviscidosis. Przegl. Epidemiol..

[9] Vissing NH, Chawes BLK, Bisgaard H (2013). Increased risk of pneumonia and bronchiolitis after bacterial colonization of the airways as neonates. Am. J. Respir. Crit. Care Med..

[10] Turk D, May J (1967). Haemophilus Influenzae. Its Clinical Importance.

[11] The Clinical and Laboratory Standards Institute. *Performance Standards for Antimicrobial Disk Susceptibility Approved Standard—9th Edition. CLSI document M02-A9* (2006).

[12] The European Committee on Antimicrobial Susceptibility Testing. *Breakpoint Tables for Interpretation of MICs and Zone Diameters. Version 3.1.*

[13] Andrzejczuk S, Kosikowska U, Chwiejczak E, Stępięń-Pyśniak D, Malm A (2019). Prevalence of Resistance to β-lactam antibiotics and bla genes among commensal *Haemophilus parainfluenzae* isolates from respiratory microbiota in Poland. Microorganisms.

[14] Barry AL, Fuchs PC, Brown SD (2001). Identification of beta-lactamase-negative, ampicillin-resistant strains of *Haemophilus influenzae* with four methods and eight media. Antimicrob. Agents Chemother..

[15] Delgado-Valverde M (2017). MIC of amoxicillin/clavulanate according to CLSI and EUCAST: Discrepancies and clinical impact in patients with bloodstream infections due to Enterobacteriaceae. J. Antimicrob. Chemother..

[16] Wienholtz NH, Barut A, Norskov-Lauritsen N (2017). Substitutions in PBP3 confer resistance to both ampicillin and extended-spectrum cephalosporins in *Haemophilus parainfluenzae* as revealed by site-directed mutagenesis and gene recombinants. J. Antimicrob. Chemother..

[17] Bork JT (2017). Impact of CLSI and EUCAST Cefepime breakpoint changes on the susceptibility reporting for Enterobacteriaceae. Diagn. Microbiol. Infect. Dis..

[18] Hinic, V., Reist, J., Schibli, U., Linnik, J. & Egli, A. Beta-lactam susceptibility in *Haemophilus influenzae*: The connection between genotype and phenotype. in *27th ECCMID* (ECCMID, 2017).

[19] Sanchez-Bautista A, Coy J, Garcia-Shimizu P, Rodriguez JC (2018). From CLSI to EUCAST guidelines in the interpretation of antimicrobial susceptibility: What is the effect in our setting?. Enferm. Infecc. Microbiol. Clin..

[20] O’Halloran C (2018). Assessment of the comparability of CLSI, EUCAST and stokes antimicrobial susceptibility profiles for *Escherichia coli* uropathogenic isolates. Br. J. Biomed. Sci..

[21] The Clinical and Laboratory Standards Institute. *Performance Standards for Antimicrobial Susceptibility Testing. 27th ed. CLSI supplement M100* (2017).

[22] European Committee on Antimicrobial Susceptibility Testing. *Breakpoint Tables for Interpretation of MICs and Zone Diameters. Version 9.0* (2019).

[23] CLSI. *CLSI M100-ED30:2020 Performance Standards for Antimicrobial Susceptibility Testing, 30th Edition* (2020).

[24] The European Committee on Antimicrobial Susceptibility Testing. *Breakpoint Tables for Interpretation of MICs and Zone Diameters. Version 10.0* (2020).

[25] Jorgensen JH, Howell AW, Maher LA (1990). Antimicrobial susceptibility testing of less commonly isolated Haemophilus species using Haemophilus test medium. J. Clin. Microbiol..

[26] Tristram S, Jacobs MR, Appelbaum PC (2007). Antimicrobial resistance in *Haemophilus influenzae*. Clin. Microbiol. Rev..

[27] Kassim A, Omuse G, Premji Z, Revathi G (2016). Comparison of Clinical Laboratory Standards Institute and European Committee on Antimicrobial Susceptibility Testing guidelines for the interpretation of antibiotic susceptibility at a University teaching hospital in Nairobi, Kenya: A cross-sectional stud. Ann. Clin. Microbiol. Antimicrob..

[28] Marchese A, Esposito S, Barbieri R, Bassetti M, Debbia E (2012). Does the adoption of EUCAST susceptibility breakpoints affect the selection of antimicrobials to treat acute community-acquired respiratory tract infections?. BMC Infect. Dis..

[29] Kosikowska U, Stępień-Pyśniak D, Ożga D, Wernicki A, Malm A (2014). Identification of Bacillus spp. colonizing the nasal mucosa of healthy adults living in the suburban area using the matrix-assisted laser desorption-ionization time-of-flight mass spectrometry (MALDI-TOF MS) system. Curr. Issues Pharm. Med. Sci..

[30] Bauer AW, Kirby WM, Sherris JC, Turck M (1966). Antibiotic susceptibility testing by a standardized single disk method. Am. J. Clin. Pathol..

[31] European Committee on Antimicrobial Susceptibility Testing. *Breakpoint Tables for Interpretation of MICs and Zone Diameters. Version 7.0* (2017).

[32] The Clinical and Laboratory Standards Institute. *Performance Standards for Antimicrobial Disk Susceptibility Approved Standard—26th edition. CLSI document M100S* (2016).

